# Initial postoperative plasticity as a predictor of mid-term stereoacuity outcome after surgery for intermittent exotropia

**DOI:** 10.1186/s12886-023-02958-6

**Published:** 2023-05-15

**Authors:** Peipei Liu, Jing Fu, Ronghan Zhang, Hang Chu

**Affiliations:** 1 Beijing Tongren Hospital, Beijing Ophthalmology & Visual Sciences Key Laboratory, Beijing Tongren Eye Center, Capital Medical University, Beijing, China; 2 Office of Academic Research, National Engineering Research Center for Healthcare Devices, Guangzhou, China

**Keywords:** Intermittent exotropia, Stereopsis, Plasticity, Prediction, Visual perception examination

## Abstract

**Background:**

Intermittent exotropia (IXT) would cause different degrees of damage to stereopsis. We aimed to introduce a visual perception plasticity score (VPPS) that reflects initial postoperative plasticity and evaluate its effectiveness in predicting the mid-term surgical outcome in IXT patients.

**Methods:**

A total of 149 patients with intermittent exotropia who underwent surgery in November 2018 and October 2019 were recruited. All subjects underwent detailed ocular examinations before and after surgery. VPPS were calculated based on visual perception examination system at one week postoperatively. Demographic, angle of deviation and stereopsis were collected and analyzed with regard to the VPPSs preoperatively and at one week, one month, three months, six months postoperatively. Predictive performances of VPPS were assessed using receiver operating characteristic (ROC) curves, the area under the curve (AUC) and cut-offs were obtained.

**Results:**

Of the 149 patients, the average deviation was 43^Δ^ at distance and 46^Δ^ at near. The average rate of normal stereopsis before surgery was 22.81% at distance and 29.53% at near. Higher VPPS was associated with preoperative better near stereoacuity (r = 0.362, p = 0.000), less angle of deviation at distance (r=-0.164, p = 0.046), and better near (r = 0.400, p = 0.000) and distant stereoacuity (r = 0.321, p = 0.000) during the early postoperative period (1 week). The areas under the curves suggested that VPPS could be an effective predictor of sensory outcome(AUC>0.6). Cut-off values of 50 and 80 were calculated for VPPS using ROC curve analysis.

**Conclusion:**

Higher VPPSs were associated with a greater possibility of stereopsis improvement in patients with IXT. VPPS is a potentially promising indicator to predict the mid-term surgical outcome of intermittent exotropia.

## Introduction

Intermittent exotropia is a common form of exotropia and is more prevalent in Asia [1], affecting up to 3.5% of the Asian population [2]. IXT is characterized by an intermittent outward deviation throughout the day. Although the etiology of IXT is largely unknown, a defect of the fusion faculty is one of the important theories, and the patients maintain normal retinal correspondence and well-established binocular function when they are ortho. Intermittent exotropia is controlled by fusional mechanisms intermittently, and it may progress to constant exodeviation with accompanying loss of stereovision.

IXT is usually treated with surgery, when exodeviation angle grows, control of IXT deteriorates, and stereopsis decreases for near and far. However, many patients still cannot restore binocular single vision even after successful surgery. D Andalib and collogues reported only 38.1% of patients regained normal stereopsis even after a perfect surgical realignment [3]. The greater the damage in stereopsis, the more difficult to recover to normal condition [4].

There has been extensive study on predicting postoperative surgical and visual perception sensory statuses, using preoperative and postoperative indicators [4–13].To date, however, few studies have been done to predict sensory outcomes [11]. It is reported that the immediate angle appears to affect surgical success after surgery [13, 14], similarly, we want to investigate whether the initial postoperative plasticity is a predictor of the mid-term sensory results after intermittent exotropia surgery.

Recently, we have developed a simple scoring method, named VPPS, to evaluate the binocular visual function of patients with IXT in the first postoperative week. Based on an evaluation system for visual and perceptual examinations, the VPPS measures perceptual eye positions (PEP) and stereopsis. It is comprehensive and easy-to-use. In this study, the following questions were answered: (1) does higher immediate postoperative plasticity result in improved sensory outcomes in the mid-term? (2) whether VPPS could be used as an indicator of mid-term positive stereoacuity in IXT patients.

## Methods

### Participants

This study adhered to the Declaration of Helsinki principles and was approved by the ethics committee of the Beijing Tongren Hospital, PRC(TRECKY2018-065). Medical records of consecutive 221 cases with IXT who underwent surgery between November 2018 and October 2019 at the Beijing Tongren Hospital were retrospectively reviewed. All surgeries were performed by a single surgeon (J.F.). Inclusion criteria were as follows:(i) subjects over 5 years of age who underwent surgery, (ii) exodeviation of 15 or more prism diopters (PD) at distance or near measured by alternate prism cover test. Exclusion criteria were as follows: (i) patients less than 5 years; (ii) history of previous strabismus or ocular surgery; (iii) with other ophthalmic diseases; (iv) with dissociated vertical deviation (DVD) or oblique muscle overaction; (v) a lack of cooperation.

### Ocular examinations

Detailed ocular examinations were performed by experienced ophthalmologists and optometrists, including the best-corrected visual acuity, eye position, ocular movement, prism cover test, stereopsis examination, manifest and cycloplegic refractions, slit lamp examination, and a fundus examination.

All patients received examinations of visual and perceptual system at 1 week postoperatively, and a VPPS was calculated. Angle of deviation and stereoacuity were collected 1 week, 1 month, 3 months and 6 months postoperatively. Measurement of strabismic angle using the prism alternate cover test (PACT) at distance (6 m) and near (1/3 m). A synoptophore (Clement Clarke Ltd., Harlow, UK) was used to determine distance stereopsis. The results of distance stereopsis were divided into normal and abnormal. The near stereopsis was examined by the Randot Stereo test (Stereo Optical Co, Inc, Chicago, Illinois). A stereoacuity ≤ 60 s of arc was considered as normal. The above procedures were repeated three times, and outliers were excluded.

### Measurement and calculation of VPPS

**Fig. 1:**
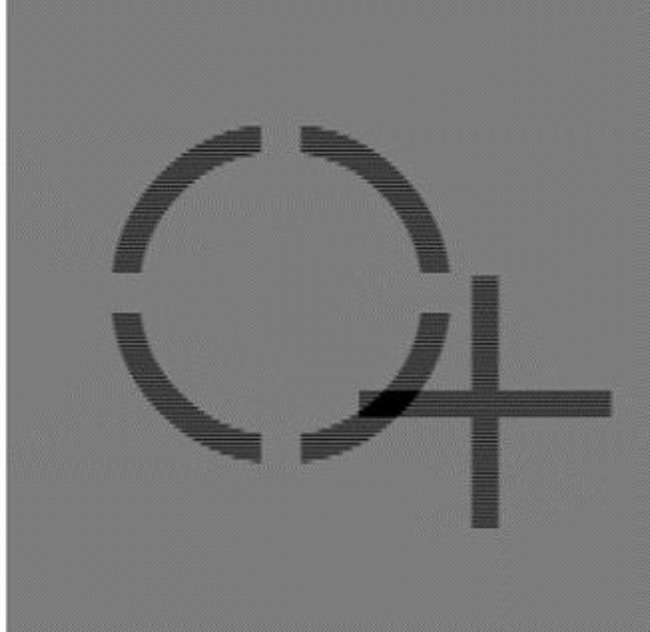
Schematic diagram of perceptual eye position examination

An examination system developed by the National Engineering Research Center for Healthcare Devices was used. The stimulating template for the examination was generated using MATLAB software. Details of visual perception examination evaluation system have been published elsewhere [15–17]and they are summarized here concisely. The measurements were conducted with 3D polarized glasses on all patients.The midpoint of the monitor was placed 80 cm away and at the same height level as the patients’ eyes. Operation and answering were done using a computer mouse or keyboard. Examinations were proceeded as follows :

(1) Perceptual eye position: using the cross-into-circle test, left eye should see a cross and right eye should see a circle (Fig. 1). Computer mice were used to place the crosses inside the circles. The minimum unit of ocular misalignment observed by this system was 1 pixel, which equals 0.04 prism.

(2) The spatial distortions: The procedure was the same as the perceptual eye position, 36 circles were evenly distributed over three circles with off-center diameters of 3.6°, 4.4°, and 5.2°, respectively. This was repeated 36 times, 12 times on each circle, and the bias pixels and standard deviations were recorded.

(3) Zero-order stereopsis [15, 18]: The E view (3 ° × 3 °) composed of random points in the central part of the distribution map has disparities of 400 ″, 300 ″, 200 ″, and 100 ″, respectively. The subject shall determine the opening direction of “E” in the image.

(4) First-order stereopsis [15, 18, 19]: E view (6 ° × 6 °) composed of random dots in the center of the stimulus map, the disparity change varies from relative zero disparity to 800 ″, upgraded at five levels: 50 ″/ms, 100 ″/ms, 200 ″/ms, 400 ″/ms, and 600 ″/ms each time, with a period of 1.2 s. The density of dynamic random dots remain unchanged. The subject decides the opening direction of “E"in the image.

(5) Second-order stereopsis [15, 18, 19]: The distribution map had a maximum of 1800 ″for crossed disparity, using a sinusoidal transform from top to bottom, the relative disparity at random dots followed a maximum of 1800′′ for uncrossed disparity and a minimum of 0 for crossed and uncrossed disparity. The subject needs to identify whether the visual target in the figure is at the peak or trough.

(6) Calculation of VPPS: First the patients were assessed by the system mentioned above, then we give them a short period (about 15 min) training. Finally, they were examined again. If the examination result is improved compared with pre-training, the score assigned by this item shall be counted; if it is not improved, the score shall be counted as 0. The scores assigned to each test are shown in Table 1. The VPPS was averaged among two parts: the perceptual eye position as well as the stereoscopic map, with a full score of 100. If the patient has reached a normal value for a test before short-term training, the assigned score is also counted.

**Table 1:** The score of visual perception examination

Visual perception examination	Score
**Perceptual eye position**	
Horizontal perceptual eye position	20
Vertical perceptual eye position	20
Standard deviation of eye position distortion	30
Average displacement of eye position distortion	30
**Stereoscopic examination**	
Zero-order stereopsis	40
First-order stereopsis	30
Second-order stereopsis	30

### Statistical analysis

Statistical analysis were performed with SPSS software (version 22.0 SPSS; Chicago, IL, USA).

Normally distributed variables were expressed as with mean ± standard deviation. Abnormally distributed variables were expressed as median (P25, P75). Categorical data were expressed as number (n) and percentage (%). An analysis of Spearman rank correlation was undertaken to determine the correlation between age at surgery, duration of disease, angle of deviation before surgery, angle of deviation at 1 week after surgery and VPPS. ROC curves were p-lotted to analyze the predictive effect of VPPS on postoperative stereopsis function recovery, and Cut-off values were also calculated using the Youden index, statistical significance is determined by a *P* value of 0.05. A chi-square test was used to evaluate the relationship between rates of normal stereopsis and VPPS.

## Results

A total of 149 patients with IXT were included in this study. The mean age of the subjects was 9.74 ± 4.27 years, and 79 (53.02%) were female. Preoperative characteristics of patients were presented in Table 2. The average VPPS was 63.09. Before surgery, the mean angle of deviation was 42.99 ± 19.36 for distance and 46.07 ± 18.83 for near. As illustrated in Table 3, the Strabismus angle reduced significantly, and sensory outcome improved after surgery.

**Table 2:** Preoperative characteristics of patients with intermittent exotropia

	Mean ± SD	Range
Age at operation (year)	9.74 ± 4.27	5–34
Interval between onset and surgery, month	43.20 ± 50.40	2-264
Onset age (year)	6.25 ± 4.30	1–26
Sex (M:F)	70:79	
Preoperative deviation (PD)		
At distance fixation	42.99 ± 19.36	15–90
At near fixation	46.07 ± 18.83	15–90
Preoperative stereopsis		
At distance (normal)	34(22.81%)	
At near (≤ 60 arcsec)	44(29.53%)	
VPPS	63.09 ± 28.16	0-100

M, male; F, female; PD,prism diopter

**Table 3:** Postoperative deviation angle and stereopsis in patients with intermittent exotropia

	Angle of deviation(PD); Range		Normal stereopsis(%)	
	At distance	At near	At distance	At near
1 week after operation	0.68 ± 2.20; 0–12	0.66 ± 2.12; 0–12	38.26%	40.27%
1 month after operation	1.56 ± 3.54; 0–20	1.51 ± 3.33; 0–20	50.34%	51.01%
3 months after operation	2.48 ± 4.04; 0–20	2.40 ± 3.99; 0–20	62.42%	59.06%
6 months after operation	4.03 ± 5.18; 0–30	3.87 ± 5.15; 0–30	65.1%	52.35%

### Correlation between visual perception VPPS and ocular examination outcomes

Significant correlations between VPPS and several parameters were found (Table 4). Higher VPPS was associated with better near stereoacuity, less angle of deviation at distance preoperatively, and better near and distant stereoacuity during the early postoperative period(1 week).

**Table 4:** Correlations between VPPS and clinical data

VPPS CorrelationCoefficient *P*		
Age at operation*	-0.157	0.057
Sex^**†**^	0.053	0.520
Interval between onset and surgery*	-0.156	0.057
Angle of deviation before surgery		
At distance*	-0.164	**0.046**
At near*	-0.124	0.130
Stereopsis before surgery		
At distance^**†**^	0.051	0.537
At near^**†**^	0.362	**0.000**
Angle of deviation at postoperative week 1		
At distance*	-0.159	0.053
At near*	-0.157	0.055
Stereopsis at postoperative week 1		
At distance^**†**^	0.321	**0.000**
At near^**†**^	0.400	**0.000**

P-values < 0.05 in bold indicate statistical significance

*Pearson correlation analysis

^†^Spearman’s rank correlation analysis

### Prediction efficacy of VPPS in IXT visual function

Patients were followed at 1 month, 3 months, 6 months postoperatively. Successful sensory outcomes was 60 s of arc. An ROC analysis was performed to test the predictive ability of VPPS. The area under ROC curve and the cut-off values were summarized in Table 5.The maximum VPPS cut-off was 77.5 and minimum was 52.5.

**Fig. 2:**
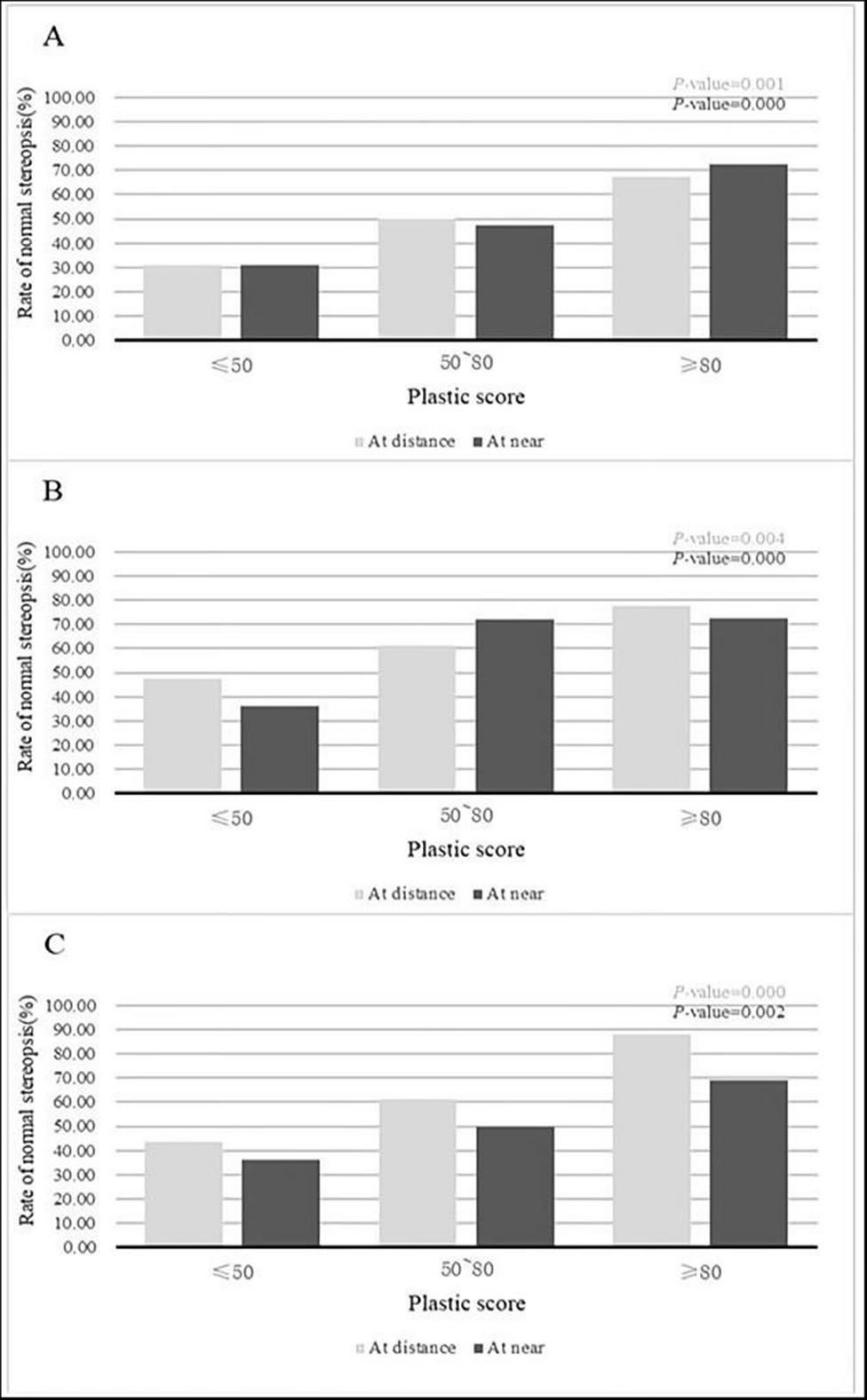
Rates of normal stereopsis at 1 month(A), 3 months(B), 6 months (C) after surgery

For computational convenience, 80 and 50 were taken as the final cut-off values to separate two different sensory outcomes. The distribution of different stereoacuity in different cut-off intervals were calculated (Fig. 2). Higher VPPS correspond to a higher likelihood of sensory success for both distance and near after surgery, which was statistically significant at different time points postoperatively (1 month, 3 months,6 months ).

**Table 5:** AUC and Cut-off values at 1month, 3months, 6months after surgery

Times	AUC	*P*	Cut-off values
Stereopsis at postoperative month 1			
At distance	0.651	0.001	62.5
At near	0.714	0.000	77.5
Stereopsis at postoperative month 3			
At distance	0.633	0.007	57.5
At near	0.720	0.000	52.5
Stereopsis at postoperative month 6			
At distance	0.738	0.000	52.5
At near	0.669	0.000	67.5

AUC, area under the curve

## Discussion

The present study found that IXT patients with a higher VPPS may have a better visual perception sensory outcome and confirmed the relationship between the postoperative week one ocular status and the month six sensory outcome. Hence, postoperative plasticity plays a key role in determining the mid-term (6 months ) sensory outcome of intermittent exotropia.

Even after successful surgical alignment in patients with IXT, many of them still have poor stereoacuity postoperatively [9, 20, 21]. One possible explanation for the lack of improvement in binocular vision is a lack of brain plasticity [15]. The goal of strabismus management is not only to align the eyes, but also to retain or regain stereopsis, and restore normal eye contact. The pursuit and desire for stereopsis make understanding of plasticity important.Trying to assess this “plasticity”, we propose the VPPS, patients with IXT were given a short period (15 min) of visual perceptual training 1week after surgery. The VPPS was defined according to the changes of perceptual eye position and zero-order, first-order, second-order stereopsis before and after training. The training of visual perception can remove obstructions in the visual channel, correct deficiency and decrease strabismus and improve stereopsis [15, 16]. Therefore, those who can be improved after short-term training indicate the possibility of continuing to restore stereopsis six months after surgery, which means higher plasticity. Perceptual eye position (PEP), as reported in previous studies [15–17], can provide a quantitative measurement of the sensory eye balance. Leske and Holmes [22] noted that true stereopsis was consistent with a horizontal deviation of up to 4PD. Fawcett et al [23] reported that patients with a residual angle of 5 PD or more after surgery may have poor stereoacuity outcome. Even small residual deviations might cause negative or low stereoacuity [11]. Therefore, precisely quantified deviation measurements are critical for predicting plasticity. This is exactly what it means to measure this PEP. Zero-order stereosopsis examination (synoptophore, random point examination, titmus) is widely used by clinicians, however, it cannot measure residual stereopsis, including first-order and second-order stereoscopic function, which are often below threshold [15].The brain distinguishes between areas that process stereoscopic functions for first-order, second-order, and zero-order multidimensional spaces, a conclusion that has been confirmed by studies in psychophysics and neurobehavioral physiology [18]. Since the calculation of the VPPS originated from PEP and stereopsis, a higher VPPS value indicates a smaller angle of deviation and a better stereoacuity. The VPPS allows comprehensive and quantitative evaluation of IXT patients. It is convenient and could reduce errors caused by subjective assessment.

Several studies [4, 6, 8, 24, 25]^−^ [12, 13]investigated the preoperative and postoperative predictors of surgical and sensory outcome after intermittent exotropia surgery, most of them focused on surgical success rate, and little research has addressed the early postoperative status in predicting restoration of stereoacuity after surgery. The present study, using VPPS, confirmed significant relationships between early postoperative status and recovery of mid-term stereopsis. In our study, much more accurate scoring has been proposed, the AUC suggested that VPPS could be an effective predictor of sensory outcome(AUC>0.6). Overall, the VPPS could provide more details of the early sensory status after IXT surgery.

Better near stereoacuity, and less angle of deviation at distance preoperatively, were associated with a higher VPPS, which means a greater chance of achievement of normal stereoacuity after surgery. These findings are in-line with those of Saxena [4] and Di-MBBS [8]. Better near and distance stereoacuity during the early postoperative period (one week) were associated with a higher VPPS. However, no significant associations between postoperative angle of deviation and VPPS were observed, indicating that the degree of deviation early after the operation may not influence the postoperative recovery of stereopsis, and neither does the successful postoperative alignment ensure full reestablishment of stereopsis.

The predictive ability of VPPS was evaluated by comparing its values among groups. We obtain two cut-off values from the ROC analysis, patients were divided into three groups. In the group of VPPS ≥ 80, about 75% of patients achieved a “normal stereopsis”, in the group of VPPS ≤ 50, this rate is only about 38%, a VPPS between 80 and 50, the rate is about 57%. The prognosis is likely to be poor if the score is less than 50, and likely favorable if the score is greater than 80. This result demonstrated that the higher the VPPS, the greater the likelihood of achieving normal stereopsis after surgery. Cutoff values of 50 and 80 can be recommended for predicting chance of stereopsis recovery.

Like most studies, this one has limitations. First, the follow-up period was only 6 months after surgery, and long-term stereopsis outcomes were not investigated. Since recurrence is common [26–28] in IXT patients, a longer follow-up period is needed to better detect fluctuations in binocular visual function. Second, a larger number of patients is required for further prospective studies to confirm the results of this study. Third, according to data from a survey [29], most responding pediatric ophthalmologists do not use a specific control score in managing intermittent exotropia. Like most investigators, we did not measure the IXT Control Score in this study. Since it is helpful to allow for more objective quantification of control in IXT, we may add other relevant information to our study in the future. Fourth, intermittent exotropia is a common childhood illness. To better understand the disease’s pathogenesis, younger children are needed. We excluded uncooperative subjects, conducted repeated examinations, and chose relatively older children with an average age of 9.74 years old. Yet, there may be inaccuracies. It is a common problem in studies of pediatric patients. In addition, this study is only a preliminary exploration and could be further refined like other scoring systems.

In conclusion, the VPPS is a useful index and could be used as a predictive factor for mid-term sensory outcomes in eyes with intermittent exotropia.In the future, we will perform more studies to determine whether the preoperative VPPS predicts postoperative stereopsis recovery, which will help determine the appropriate timing for surgery.

## Data Availability

The datasets used and/or analysed during the current study available from the corresponding author on reasonable request.
